# Editorial: Next-generation quantitative and synthetic biology: High-sensitivity, high-accuracy, and digital approaches

**DOI:** 10.3389/fbioe.2023.1146729

**Published:** 2023-02-06

**Authors:** Burak Okumus, Juan M. Pedraza, Somenath Bakshi

**Affiliations:** ^1^ XCellCure, St. Louis, MO, United States; ^2^ Department of Physics, University of Los Andes, Bogotá, Colombia; ^3^ Department of Engineering, University of Cambridge, Cambridge, United Kingdom

**Keywords:** synthetic & systems biology, microfluidics, microbiome, single molecule, antibiotic resisitance, macromolecular crowding, organ on a chip, microphysiological systems

The articles in this collection describe, review, and utilize recent developments of technologies, which enable us to tackle various practical challenges, from healthcare to bioproduction. The Research Topic thus covers a wide range of complexities, from molecules to cells to communities of cells, providing an outlook for the recent advances in studying, analyzing and engineering of biological systems across scales (Figure 1).

**FIGURE 1:**
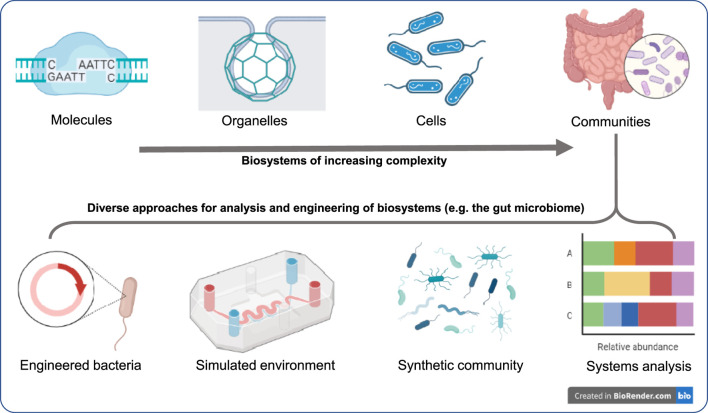
Understanding and engineering biological systems across scales: This article Research Topic represents new results and reviews on approaches that range from individual molecules and organelles to cells and their communities. The Research Topic also demonstrates that at any of these scales, multitude of approaches can be used for analysis and bottom-up engineering, which lays the groundwork for detailed understanding and designing practical applications. For example, to understand the microbiome and to devise technologies to address its dysbiosis, in addition to systems analysis of the native gut, engineered systems and methods for engineering synthetic communities to simulate the interaction, or simulating the gut environment (organ-on-a-chip type microfluidic devices), or utilizing synthetic diagnostic bacteria can provide unprecedented insight. Further attesting to their promise, the Food and Drug Administration (FDA, 2022) recently identified such microphysiological systems as one of the priority areas [Advancing Alternative Methods at FDA] to foster innovative products.

Setting the stage all the way from the basic building-blocks of life processes, Kang et al. extensively reviewed how watching individual molecules shed light on the mechanisms that the cells employ to maintain their genomes. The surveyed literature of DNA-repair, employing fluorescence microscopy, exemplify how single-molecule methods unveil detailed molecular pathways, otherwise inaccessible *via* traditional bulk methods. For investigating biological systems with resolution beyond the diffraction limit (which was considered a fundamental, unsurmountable physical barrier for over a century), single-molecule imaging enabled super-resolution microscopy, and was awarded with the Nobel Prize in 2014. Another super-resolution modality, called the “structured illumination microscopy” (SIM), uses a known pattern of excitation beams to generate super-resolution images, and enables temporal resolution higher than that of its Nobel-winning counterpart. Akatay et al. used SIM to investigate the major endocytotic route in cells, i.e., formation of clathrin-coated vesicles (or pits, henceforth abbreviated as CPs), under mechanical tension. By simultaneous utilization of various modes of SIM with varying imaging depths (namely, TIRF-SIM and the recently developed GI-SIM (Guo et al., 2018), they were able to unambiguously rule out the “flat-to-curved transition” model for curvature generation and elucidate the details of the internalization of CPs. Moreover, they also observed that external perturbations that increase in the membrane tension, stimulated the formation of “giant coated pits” (GCPs).

While the mechanical properties of native membranes dictate the release of endocytotic vesicles (such as CPs and GCPs), their complex and heterogeneous compositions have motivated the development of controllable model-membrane systems, such as synthetic lipid vesicles (a.k.a liposomes). Liposomes have been extensively used as lipid-membrane mimics—e.g. to investigate how various lipid compositions, reconstituted membrane proteins, or the ambient conditions affect vesicles’ biological and physical properties (Pick et al., 2018). In this Research Topic, Quinn et al. present an investigation that for the first time explores how the extravesicular crowding affect the morphology of liposomes, by employing a combination of complimentary methods: single-vesicle imaging, single-molecule FRET, dynamic light scattering, and fluorescence lifetime analysis. They found that various crowding agents induced liposome compaction, and such structural alterations could be reversible or irreversible depending on crowder size. Regulation of liposome architecture due to molecular crowding could have implications for neurotransmitter release and endosomal entry/escape of viruses. On the applied side, such regulation can enable fine control of liposome-mediated drug delivery or to use the liposome morphology as a mechanosensitive readout to spatially map the extent of crowding within cells or tissues.

At a higher level than individual molecules and molecular assemblies, studies on individual cells have gained traction in the past decades. Single-cell studies, while typically requiring a lower resolution, still pose an important challenge for maintaining physiologically relevant imaging conditions over extended periods. Living cells alter their environment while responding to it, by consuming the nutrients, releasing byproducts and signaling molecules. Simultaneously, their numbers also vary (due to division or death), causing both the ambient conditions and cell densities to change over time. Allard et al. highlighted advancements based on the “mother machine”, a microfluidic device that is extremely powerful in sustaining cell cultures under growing conditions, while observing them with single-cell resolution over time periods that are otherwise unattainable. Microfluidic platforms offer unmatched opportunities for cultivating cell cultures, even replicating tissue architectures. Such systems, sometimes called “organ-on-a-chip”, are conducive for co-culturing endothelial, epithelial, and dendritic cells together with macrophages and bacteria, and can therefore serve as tractable models for studying the complex interplay between microbiome and host (Siwczak et al., 2021) under well-defined conditions and on-demand perturbations.

Single-cell measurements within microfluidic devices have also advanced antibiotic sensitivity testing (AST)—allowing rapid identification of pathogens and their susceptibility towards antimicrobial drugs (Baltekin et al., 2017). Here Farid et al. describe the development of an unconventional technology [Rapid Ultrasensitive Detector, (RUSD)] for rapid and label-free detection of bacterial cells in clinical settings. This new method of AST uses hollow silica fibres—optofluidic channels that simultaneously function as lightguides and sample-reservoirs. RUSD is extremely sensitive in detecting changes in the light intensity, and therefore can detect the presence of bacteria at very low densities despite their minute light scattering. As a result, the bacterial growth (or lack thereof) in the presence of antibiotics can quickly be assessed without having to wait for cells to reach high densities for their detection, thus circumventing a major problem of conventional AST. State-of-the art technologies that can minimize the turnaround time of AST are critical to keep the emergence and spread of resistant mutants of pathogenic species at bay by reducing the use of ineffective antibiotics.

Rapid and accurate detection of cells in a culture could also be used for applying feedback control to their abundance. The ability to quantify cell densities while identifying different strains paves the way for controlling composition of a community of cells. Lee et al. review how computer-enabled feedback-control using automated measurements and external inputs (termed as “cybergenetic control”) can be used to modulate the composition of synthetic microbial communities. They focus on the applications of cybergenetic control, in the context of bioproduction, where consortia of microbes open up the possibilities of reducing production burden (a crucial objective to prevent the producer to be outcompeted) through division of labor, allow the use of cheaper or more complex substrates, and provide extra robustness towards fluctuations in environmental conditions. By measuring the state of a community (e.g. *via* real-time microscopy) and applying external inputs (e.g. inducers or antibiotics), cybergenetics can drive the system towards a desired reference (e.g. a certain composition of the constituting strains). Furthermore, cybergenetic approaches can prove invaluable to explore complex natural microbial communities, from applications in agriculture of biomedicine, for instance to provide educated guesses for what effects external perturbations (e.g. probiotics or pesticides) would produce.

Further moving up in complexity from the synthetic microbial communities, comprised of well-defined, limited number of species, Robinson et al. review the latest methods using microbial synthetic circuits for spatially-precise diagnostics and therapy within the complex, dynamic and spatially-heterogeneous environment of the mammalian gut. As the role of the microbiome towards our health is increasingly becoming obvious—from predicting the efficacy of drugs (Haiser et al., 2013) to COVID-19 severity (Yeoh et al., 2021)—the article highlights advances in methods to employ engineered bacterial strains for the detection of target pathogens and release of therapeutics to combat them, while maximizing effectivity and minimizing collateral damage (e.g. dysbiosis, local or systemic inflammatory side-effects). However, the authors also highlight the challenges towards engineering such biosystems, which arise from unpredictable context-dependent effects. Engineered biological systems contain various parts that interact with each other, their chassis, and the environment where the entire engineered system is placed in. Moschner et al. present an organized approach of considering the various contextual factors that affect the performance of any engineered biological system. They propose a conceptual framework called “context matrix” where such contextual input factors are categorized along three dimensions: construct, host, and environment. Understanding the position of an engineered system in this multidimensional space can help understand the failure modes and design better systems. The authors have created a database for this matrix on GitHub—and welcome community contribution towards its enrichment—which could in the future assist context-aware biodesign for better prediction and robust performance.

While the articles in this Research Topic cover a wide variety of scales, techniques and systems, they all illustrate how the development of ever more precise and controllable ways of measuring and manipulating biological systems must be combined with ways of determining emergent effects and connections across scales to both decipher and design biological systems. In physics, there is a longstanding philosophical dispute between the particle physics and condensed matter subfields on whether the best way to advance our understanding of the Universe is to look for ever smaller scales and more detail or to better understand emergent properties of collectives. This Research Topic shows that in biology, we can and must do both.

## References

[B1] Baltekin O. Boucharin A. Tano E. Andersson D. I. Elf J. (2017). Antibiotic susceptibility testing in less than 30 min using direct single-cell imaging. Proc. Natl. Acad. Sci. U. S. A. 114, 9170–9175. 10.1073/pnas.1708558114 28790187 PMC5576829

[B2] FDA (2022). Advancing alternative methods at FDA. Available at: https://www.fda.gov/science-research/about-science-research-fda/advancing-alternative-methods-fda .

[B3] Guo Y. Li D. Zhang S. Yang Y. Liu J. J. Wang X. (2018). Visualizing intracellular organelle and cytoskeletal interactions at nanoscale resolution on millisecond timescales. Cell. 175, 1430–1442.e17. 10.1016/j.cell.2018.09.057 30454650

[B4] Haiser H. J. Gootenberg G. B. Chatman K. Sirasani G. Balskus E. P. Turnbaugh P. J. (2013). Predicting and manipulating cardiac drug inactivation by the human gut bacterium eggerthella lenta. Science 341, 295–298. 10.1126/science.1235872 23869020 PMC3736355

[B5] Pick H. Alves A. C. Vogel H. (2018). Single-vesicle assays using liposomes and cell-derived vesicles: From modeling complex membrane processes to synthetic biology and biomedical applications. Chem. Rev. 118, 8598–8654. 10.1021/acs.chemrev.7b00777 30153012

[B6] Siwczak F. Loffet E. Kaminska M. Koceva H. Mahe M. M. Mosig A. S. (2021). Intestinal stem cell-on-chip to study human host-microbiota interaction. Front. Immunol. 12, 798552. 10.3389/fimmu.2021.798552 34938299 PMC8685395

[B7] Yeoh Y. K. Zuo T. Lui G. Zhang F. Liu Q. Li A. Y. (2021). Gut microbiota composition reflects disease severity and dysfunctional immune responses in patients with COVID-19. Gut. 70, 698–706. 10.1136/gutjnl-2020-323020 33431578 PMC7804842

